# Difficult intubation in ENT patient: Simultaneous videolaryngoscopy with flexible bronchoscopy. A combined approach. Case report

**DOI:** 10.1016/j.ijscr.2024.109345

**Published:** 2024-02-04

**Authors:** Gaetano Ottoveggio, Barbara Verro, Maria Lapi, Francesco Tarantino, Giovanna Beccia, Carmelo Saraniti

**Affiliations:** a Unit of Anesthesia, Intensive Care, and Emergency, Department of Surgical, Oncological and Oral Science (Di.Chir.On.S.), University of Palermo, Italy; b Unit of Otorhinolaryngology, Department of Biomedicine, Neuroscience and Advanced Diagnostic (BIND), University of Palermo, Palermo, Italy; c Unit of Anesthesia, Intensive Care, and Emergency, Department of Emergency and Neuroscience, Trauma Center, Villa Sofia Cervello United Hospitals, Palermo, Italy; d Unit of General Surgery and Transplant Anesthesia, IRCCS A. Gemelli University Polyclinic Foundation, Sacred Heart Catholic University, Rome, Italy

**Keywords:** Intubation, Difficult intubation, Bronchoscopy, Laryngoscopy, Airway management, Hybrid technique

## Abstract

**Introduction:**

Difficult intubation is the situation when a skilled anesthetist has difficulties to manage airway using face mask, laryngoscopy, supraglottic device, tracheal intubation, surgery. Videolaryngoscope and flexible fibroscope (FFS) represent valid alternatives for difficult airway management, with some limitations. However, literature lacks of studies about the efficacy of the combined use of videolaryngoscope and FFS.

**Case report:**

We report a case of a man, with glottic lesion, who needs surgery under general anesthesia. Anesthesiologic pre-operative evaluation revealed that he's a difficult intubation case. So, in a supine position, intubation was performed on first attempt by videolaryngoscope combined with FFS. On post-op, no signs of injuries due to intubation have been found.

**Discussion:**

In 2022, the American Society of Anesthesiologists defined the guidelines to manage difficult intubation: based on patient’ anatomical and clinical feature and anesthetist’ skills, several intubation procedures could be used. Each procedure has pros and cons.

**Conclusion:**

It's the first case of anticipated difficult intubation in adult man that was intubated under general anesthesia by using videolaryngoscope combined with FFS. We demonstrated that this procedure is safe and useful in case of difficult airway and recommended in case of laryngeal lesions that hinder the visualization of glottic plane.

## Highlights

• Videolaryngoscope and flexible fibroscope (FFS) are tools for difficult intubation.
• Intubation technique is based on patient ‘feature and anesthetist’ skills.
• Anticipated difficult intubation can be managed by videolaryngoscope and FFS.

## 1 Introduction

Difficult intubation is defined as the situation when a skilled anesthetist has difficulties to manage airway using face mask, laryngoscopy, supraglottic device, tracheal intubation and/or surgery (e.g., cricothyrotomy, tracheostomy and extracorporeal membrane oxygenation – ECMO) [1,2]. In 2022, American Society of Anesthesiologists updated the guidelines in case of difficult airway management [1]. In particular, in case of anticipated difficult intubation, these guidelines suggest different possible approaches: awake or anesthetized tracheal intubation by using rigid laryngoscopic blades, videolaryngoscopes, flexible fibroscope, supraglottic airway devices, rigid bronchoscope, combination of these techniques, or invasive procedures such as cricothyrotomy, tracheostomy and ECMO. However, as reported also in this paper, the choice of intubation technique depends on patient in terms of age, BMI, cooperation, comorbidities, on surgery to perform and on skills of anesthetist. Indeed, each procedure showed a high success rate. Several studies demonstrated that videolaryngoscopy ensures better intubation than direct laryngoscopy: enhanced view of larynx, higher success rate of intubation, higher intubation rate at first attempt [[3], [4], [5], [6]]. Intubation by using flexible fibroscope (FFS) represents a valid alternative in case of difficult airway management given that this technique allows to view the larynx, to avoid trauma, to correctly direct the bronchoscope and so the endotracheal tube (ET) into the trachea. Studies about this procedure report a success rate of intubation ranging from 75 % to 100 % [[7], [8], [9]].

However, the current literature lacks of studies about the efficacy of the combined use of videolaryngoscope and FFS [[10], [11], [12]], above all under general anesthesia (GA) [13,14].

So, based on these assumptions, we reported a case of a man affected by glottic lesion with anticipated difficult airway management that was intubated by using videolaringoscope and FFS simultaneously under general anesthesia. This work has been reported in line with the SCARE criteria [15].

## 2 Case report

We report a case of a 51-year-old man, 1.72 cm tall and weighing 87 Kg, with a suspected glottic cancer that underwent transoral laryngeal microsurgery (TLM) under general anesthesia in order to perform biopsies for the histological diagnosis. He's a smoker of 25 cigarettes per day for 35 years, without alcohol habits. He doesn't report history of previous surgeries under general anesthesia. He suffers from gastroesophageal reflux disease (GERD) and obstructive sleep apnea syndrome (OSAS). During the pre-operative period, he underwent blood tests, neck and chest computed tomography with contrast medium, cardiologic and pneumological visits that didn't reveal any disease or abnormalities. The pre-operative fiber optic laryngoscopy showed a suspected lesion that involves the whole left vocal cord – from the anterior commissure to the vocal apophysis of the arytenoid cartilage – and extended to the left Morgagni's ventricle and to the anterior third of right vocal cord. Anesthesiologic pre-operative evaluation revealed: overweight condition (BMI 29.4 kg/m2), normal dentition, no trismus, interincisive opening distance greater than 3 cm, thyromental distance lesser than 6 cm, with good cervical mobility, Mallampati III, ASA [16] III. Based on this evaluation, the patient was classified as a difficult intubation case.

Once in the operating room, in a supine position on the operating table, vital signs of patients were monitored: peripheral oxygen saturation (SpO2), non-invasive blood pressure (NIBP) on his left arm, heart rate (HR), and cardiac activity by electrocardiogram (ECG). First, the patient was pre‑oxygenated for 5 min and premedicated by administration of intravenous midazolam 2 mg and fentanyl 100 μg. Then, GA was induced by administration of intravenous propofol 200 mg and rocuronium bromide 100 mg.

**Fig. 1:**
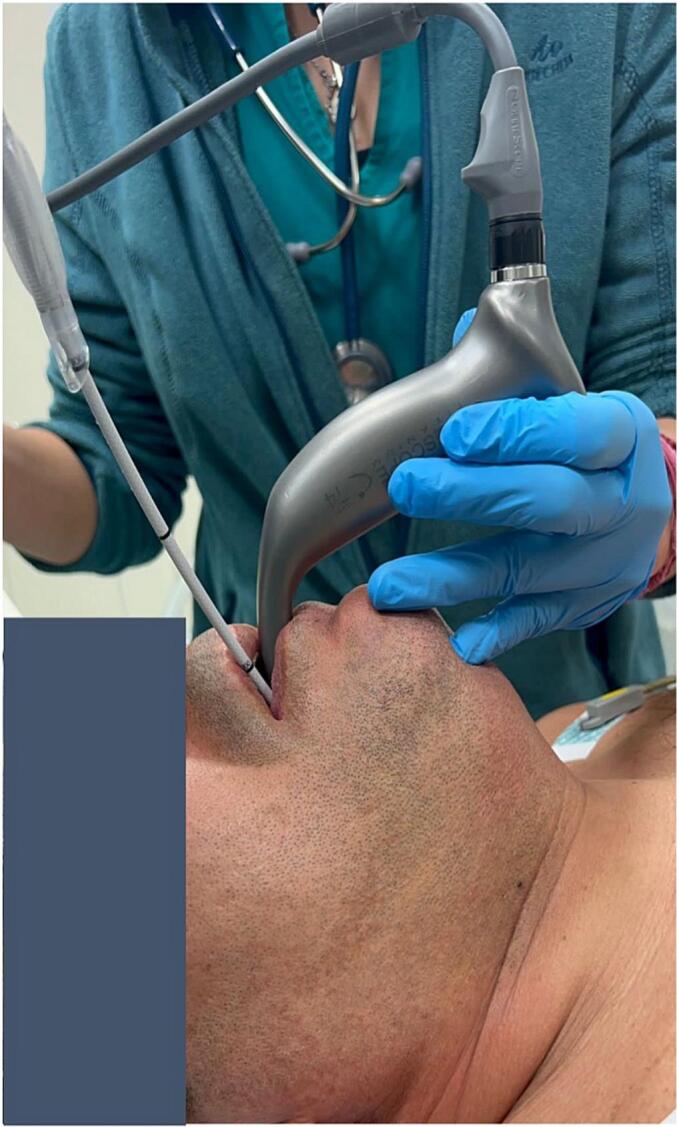
External view of combined approach: videolaryngoscope and flexible fibroscope.

So, based on the pre-operative evaluation of the airway and on the type of surgery (transoral laryngeal laser surgery), a combined technique of intubation was performed by two skilled anesthetists: video laryngoscopy combined with FFS (Fig. 1).

**Fig. 2:**
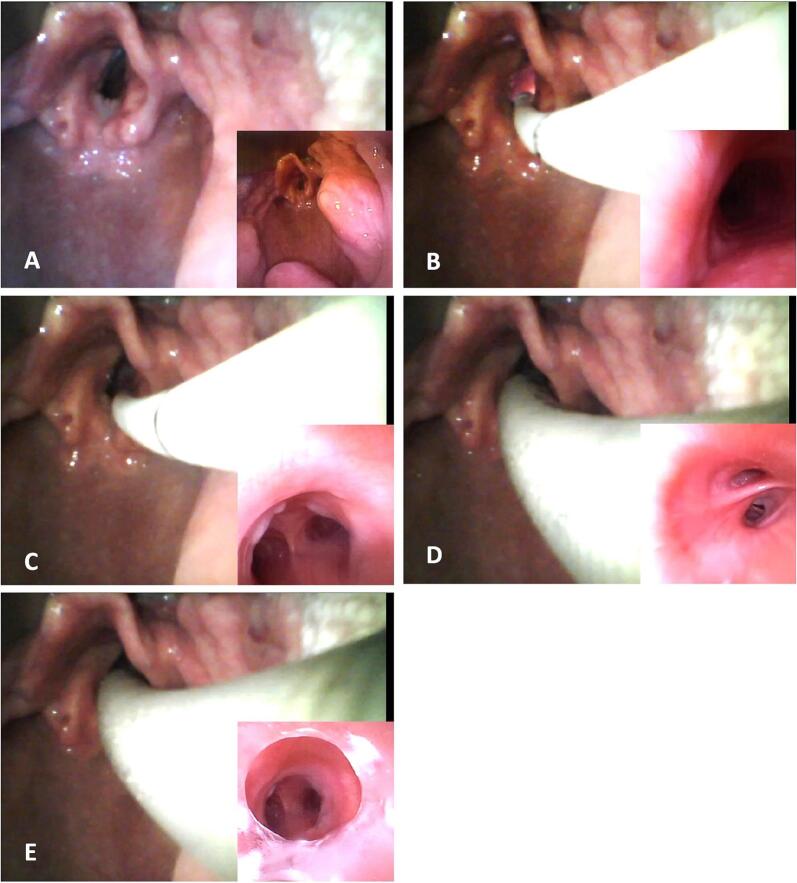
Combined technique (bigger screen by videolaryngoscope, smaller screen by flexible fibroscope (FFS)): double vision of the laryngeal aditus (A); FFS beyond the vocal cords (B); FFS above the tracheal bifurcation (C); endotracheal tube (ET) beyond the glottic plane (D); check of the correct position of the ET by FFS (E).

In particular, one anesthetist held the videolaryngoscope in place to keep the mouth and the oropharynx open and to facilitate the correct direction of the fibroscope. So, the second anesthesist introduced the fibroscope orally and, thanks to the dual vision on the screen (of videolaryngoscope and fibroscope), reached the glottis and the trachea avoiding hitting the anatomical structures and the ulcerated vocal cord lesion that was at high risk of bleeding. Once in the trachea, the fibroscope is placed immediately above the tracheal bifurcation to prevent the displacement and exit of the fibroscope from the trachea during the intubation maneuver. Indeed, the ET – previously loaded on the fibroscope – has been slid down to the trachea, under dual vision. At the end of the procedure, with the tip of fibroscope, the anesthesist has checked the correct position of the ET (Fig. 2).

Using this approach, intubation succeeded at the first attempt: the patient was intubated with a 5.5 mm armored endotracheal tube. Ventriculotomy and multiple glottic biopsies were performed by the ENT surgeon. At the end of surgery, a safe extubation was performed according to the international guidelines [17].

During post-operative, the patient didn't report dysphagia or dyspnea and didn't show any sign of mandibular subluxation. On post-op day 1, no signs of injuries or lesions due to intubation have been found during oropharyngoscopy and fiberoptic pharyngo-laryngoscopy, except for the surgical site that is the glottis.

## 3 Discussion

In 2022, the American Society of Anesthesiologists defined the last version of practice guidelines to manage difficult intubation [1]. They stated an algorithm for adult patients: based on patient’ anatomical and clinical feature and anesthetist’ skills, several intubation procedures could be used. Each one has pros and cons. Among the alternatives of intubation, the combination techniques are described, e.g., laryngoscopy (video or direct) with supraglottic device or FFS or guide catheter. We report a case of anticipated difficult intubation that was managed with hybrid technique: videolaryngoscope and FFS. Reviewing the current literature, we found few manuscripts about this approach: most describe cases of awake intubation [[10], [11], [12]]. Utada et al. showed a clinical video of combined intubation in awake patient [10]. The video shows that this procedure in awake patient result difficult; indeed, the presence of pharyngeal reflexes, cough reflex and the adduction of the vocal cords as defense mechanisms make it difficult to reach the trachea with the fibroscope despite double vision, prolonging intubation time. As reported also by Xue et al., a careful patient's selection for awake intubation is mandatory [11]. Only two studies report this hybrid technique under general anesthesia. In their letter to editor, Greib et al. describe 16 cases of intubation by combining videolaryngoscope and FFS [13]. However, the authors specify that none of them was a difficult intubation. On the contrary, our patient was an anticipated difficult airway management, so intubation at first attempt was of great importance. Anyway, in neither case complications were found in the post-operative. The other study about combined intubation approach under general anesthesia concerns the pediatric population (while our patient was 51 years old) [14]. They demonstrated that this technique has a high success rate with few complications. So, they recommend keeping this technique in mind in the algorithm of difficult intubations, without forgetting the technical difficulty of the procedure. Indeed, as reported in our case report, this approach needs two anesthetists, of which at least one must be an expert fibroscopist. Moreover, in our case report, in addition to the issue of difficult intubation, the patient had a suspected and ulcerated-bleeding glottic lesion; so, it was equally important not to touch and to hit the vocal cords to avoid bleeding. Actually, this event could affect the assessment of the lesion by diagnostic intra-operative narrow-band imaging (NBI) [18] and make the surgery more difficult.

As reported by the authors who performed this technique, it has many advantages: under videolaryngoscope vision, it is easier to direct the tip of the fibroscope avoiding injuries, above all on arytenoid cartilages that are subject to post intubation granulomas and subsequent dyspnea. In performing this procedure, a pearl is the use of armored endotracheal tube that is softer and less traumatic that non-armored ET. Also, this caution can reduce the risk of arytenoids injuries.

## 4 Conclusion

The take home message is that this hybrid technique turns out to be useful in case of laryngeal lesions (supraglottic and/or glottic lesions) that modify the anatomy of hypopharynx and larynx and so, impair the visualization of glottic air space. On one side, the videolaryngoscope provides a panoramic view of pharynx and larynx, however it doesn't allow to view the glottic plane if it's covered by the lesion. On the other side, due to its flexibility and tip orientation, the FFS allows to visualize the glottis air space even if it's reduced by the lesion. However, it has the drawback of not providing a panoramic view making its maneuverability and orientation difficult, above all in the presence of secretions, blood and tongue base that occupies the hypopharynx.

As far as we know, we reported the first case of anticipated difficult intubation in adult man affected by an ulcerated glottic lesion that was intubated successfully under general anesthesia on the first attempt by using combined videolaryngoscope and flexible fibroscope. We demonstrated that this procedure is safe and useful in case of difficult airway and recommended in case of laryngeal lesions that hinder the visualization of glottic plane. Obviously further studies with larger casuistry need to validate and promote this technique; it's crucial to never stop looking for new and more effective procedures.

## Ethical approval

We acquired the consent for publication from patient but we don't require ethical approval since it is an anonymous case report.

## Funding

This research did not receive any specific grant from funding agencies in the public, commercial, or not-for-profit sectors.

## Author contribution

Barbara Verro: Data Curation, Writing - Original Draft;

Gaetano Ottoveggio: Conceptualization, Methodology, Supervision;

Maria Lapi: Data Curation, Visualization;

Francesco Tarantino: Conceptualization, Writing - Original Draft;

Giovanna Beccia: Validation, Visualization;

Carmelo Saraniti: Methodology, Validation, Supervision

## Guarantor

Barbara Verro

## Note

Written informed consent was obtained from the patient for publication of this case report.

## Conflict of interest statement

The authors declare no conflict of interest.
